# Non-Myeloid Cells are Major Contributors to Innate Immune Responses via Production of Monocyte Chemoattractant Protein-1/CCL2

**DOI:** 10.3389/fimmu.2013.00482

**Published:** 2014-01-07

**Authors:** Teizo Yoshimura, Carole Galligan, Munehisa Takahashi, Keqiang Chen, Mingyong Liu, Lino Tessarollo, Ji Ming Wang

**Affiliations:** ^1^Laboratory of Molecular Immunoregulation, Cancer and Inflammation Program, Center for Cancer Research, National Cancer Institute, Frederick, MD, USA; ^2^Mouse Cancer Genetics Program, Center for Cancer Research, National Cancer Institute, Frederick, MD, USA

**Keywords:** chemokines, inflammation, innate immunity, myeloid cells, gene knockout mice

## Abstract

Monocyte chemoattractant protein-1 (MCP-1)/CCL2 is a chemokine regulating the recruitment of monocytes into sites of inflammation and cancer. MCP-1 can be produced by a variety of cell types, such as macrophages, neutrophils, fibroblasts, endothelial cells, and epithelial cells. Notably, macrophages produce high levels of MCP-1 in response to proinflammatory stimuli *in vitro*, leading to the hypothesis that macrophages are the major source of MCP-1 during inflammatory responses *in vivo*. In stark contrast to the hypothesis, however, there was no significant reduction in MCP-1 protein or the number of infiltrating macrophages in the peritoneal inflammatory exudates of myeloid cell-specific MCP-1-deficient mice in response to i.p injection of thioglycollate or zymosan A. Furthermore, injection of LPS into skin air pouch also had no effect on local MCP-1 production in myeloid-specific MCP-1-deficient mice. Finally, myeloid-specific MCP-1-deficiency did not reduce MCP-1 mRNA expression or macrophage infiltration in LPS-induced lung injury. These results indicate that non-myeloid cells, in response to a variety of stimulants, play a previously unappreciated role in innate immune responses as the primary source of MCP-1.

## Introduction

Chemokines play a pivotal role in guiding leukocyte trafficking during inflammatory responses (1). Although this is considered their primary function, chemokines also control the organization of the entire hematopoietic/lymphopoietic system, including the regulation of stem cell maturation, the formation of secondary lymphoid tissues, and angiogenesis (2–5). Moreover, chemokines and their receptors are intimately involved in the orchestration of inflammatory responses, in the pathogenesis of acquired immunodeficiency syndrome (6) and the progression of cancer (7). Therefore, they are considered to be important potential therapeutic targets in these diseases (8).

Some chemokines, such as stromal cell-derived factor 1/CXCL12, are constitutively produced by restricted cell types, such as stromal cells; however, production of many chemokines is induced in multiple cell types upon tissue injury (9). Monocyte chemoattractant protein-1 (MCP-1) is a potent monocyte chemoattractant that also attracts T cells, NK cells, and dendritic cells (10). It is produced by a variety of cell types, including macrophages, endothelial cells (ECs), epithelial cells, and neutrophils, in response to proinflammatory stimuli (11). Macrophages have been demonstrated to produce MCP-1 at many human and animal disease sites; thus, they are considered to be an important cellular source of MCP-1 and contribute to the further recruitment of monocytes, T cells, and DCs during inflammatory responses (12, 13). However, it remains unclear whether, in a complex environment of injured tissue, other cell types also produce significant levels of MCP-1. Determination of the exact cell types producing MCP-1 in diseases and the mechanisms by which they produce MCP-1 may allow us to target those cells for effective inhibition of its production.

To determine the precise cellular source of MCP-1 in immune responses, we generated MCP-1^flox/flox^ mice for tissue-specific deletion of this chemokine. In the present study, we used myeloid cell-specific MCP-1-deficient mice to evaluate whether myeloid cells, such as neutrophils and macrophages, are the main source of MCP-1 in innate immune responses. Here we report that in contrast to the original hypothesis, myeloid cell-specific MCP-1-deficiency did not reduce MCP-1 production in experimental mouse peritonitis, skin air pouch, or LPS-induced lung injury. Thus, non-myeloid cells are major MCP-1 producers and play a previously unappreciated role in the development of innate immune responses.

## Materials and Methods

### Mice

The generation of MCP-1 foxed mice (MCP-1^flox/+^) mice was previously described (14). MCP-1^flox/+^ mice was backcrossed to WT C57BL/6Ncr mice (Charles River, Frederick, MD, USA) for 10 generations, and the resulting mice were then crossed to LysMCre mice (15) on a C57BL/6 background to generate LysMCre+, MCP-1^flox/flox^ (JAX Stock No. 023347, B6;129-Ccl2 <tm1Tyos >/J). All experimental protocols for this study were approved by the Frederick National Laboratory for Cancer Research Animal Care and Use Committee, Frederick, MD, USA.

### Induction of TG- or zymosan A-induced peritonitis

Eight- to twelve-week-old mice were intraperitoneally injected with 1 ml of sterile, 3% TG broth (Difco Laboratories, Detroit, MI, USA) or 0.5 ml of 400 μg/ml zymosan A (Sigma-Aldrich, St. Louis, MO, USA). The mice were sacrificed 4 h after injection to measure MCP-1 concentration or 4 days to evaluate macrophage infiltration. Peritoneal exudates were harvested by peritoneal lavage using 5 ml cold PBS. The concentration of peritoneal exudates cells (PEC) was counted using a hemocytometer under microscope. Cells were applied to microscope slides, using a cytospin centrifuge (Shandon), and stained with Diff-Quick (Baxter Healthcare Corp.), and differential cell counts were obtained by morphological analysis. The number of macrophages was calculated, using the total cell number and the percentage of macrophages in the same sample.

### Air pouch model

Air pouches were raised on the dorsum of 6- to 8-week-old mice as described previously (16). Mice with a well-formed air pouch were randomized into groups. Each mouse was given an injection with 1 ml endotoxin-free PBS alone or PBS containing 1 mg LPS (Sigma-Aldrich) into the air pouches. Four hours after injection, mice were euthanized by CO2 asphyxiation, and cells in the air pouches were harvested with 2 ml PBS with heparin.

### LPS-induced lung injury

Mice were placed in a Mass Dosing Chamber (PLY5000, Buxco Research Systems, Wilmington, NC, USA) connected to an Aerogen Aeroneb nebulizer in a ventilated biological hood. LPS (100 or 1000 μg/ml in 10 ml PBS) was added to the nebulizer and aerosol was produced. Mice were exposed to LPS aerosol for 30 min, and then returned to cages. Four or 24 h after LPS exposure, mice were euthanized by CO2 and bronchoalveolar lavage fluids (BALFs) were collected by injecting 1 ml PBS into the lung through trachea.

### Northern and southern blotting

Northern blot and southern analyses were performed as described (14, 17). Filters were hybridized at 42°C overnight in 50% formamide, 5× SSC, 5× Denhardt’s solution, 50 μg/ml sheared-denatured salmon sperm DNA, 1% SDS, and l × 10^6^ dpm/ml of ^32^P-labeled cDNA probe (Perkin Elmer, Cambridge, MA, USA). Filters were washed twice with 2× SSC, 0.5% SDS at room temperature for 15 min and 0.1× SSC, 0.5% SDS at 60°C for 30 min prior to autoradiographic exposure.

### ELISA

The concentrations of MCP-1 were measured in the Lymphokine Testing Laboratory, Clinical Services Program, SAIC-Frederick, Frederick, MD, USA with an ELISA kit specific for mouse MCP-1 (R&D Systems).

### Statistical analysis

Statistical analysis was performed by Student’s *t*-test or Log-rank (Mantel–Cox) test, using the GraphPad Prism, Version 4 and 5, GraphPad Software, San Diego, CA, USA. A value of *p* < 0.05 was considered to be statistically significant.

## Results

### Generation of myeloid cell-specific MCP-1-deficient mice

**Figure 1:**
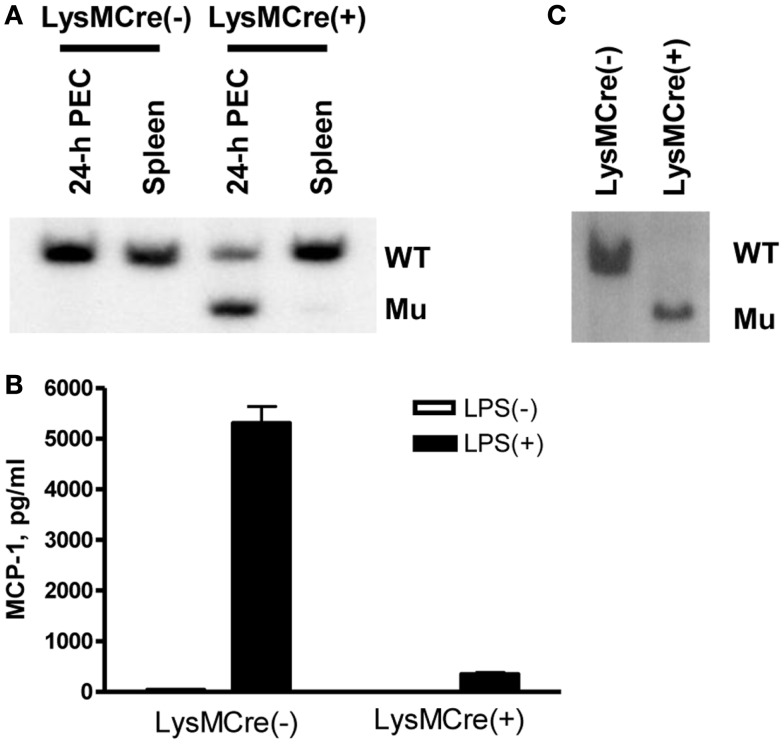
**Generation of myeloid cell-specific MCP-1-deficient mice**. **(A)** Genomic DNA was isolated from 4-day TG-induced PEC or spleen of LysMCre^−^, MCP-1^flox/flox^ or LysMCre^+^, MCP-1^flox/flox^ mice. The DNA were digested with *Pst*I and subjected to Southern blotting for the presence of WT or mutant (Mu) allele. **(B)** 1.8 × 10^6^ PEC from LysMCre^−^, MCP-1^flox/flox^ or LysMCre^+^, MCP-1^flox/flox^ mice were cultured in 1 ml medium for 24 h in the absence or presence of 100 ng/ml LPS. The concentration of MCP-1 in the culture supernatants was measured by ELISA. Data is presented as the mean ± SD obtained with cells from three mice. **(C)** PEC isolated 4 days after TG injection were incubated in tissue culture plates at 37C overnight, non-adherent cells were removed, and then adherent cells were lysed to obtain genomic DNA. The DNA were digested with PstI and then subjected to Southern blotting for the presence of WT or mutant (Mu) allele.

Myeloid cell-specific MCP-1-deficient mice were generated by crossing MCP-1^flox/flox^ mice to LysMCre^+^MCP-1^flox/flox^ mice. We first examined the efficiency of MCP-1 gene deletion in myeloid cells. As shown in Figure 1A, there was an approximately 70% reduction in the amount of WT MCP-1 allele in TG-induced PEC of LysMCre^+^, MCP-1^flox/flox^ mice by Southern blotting, and approximately 90% reduction in MCP-1 concentration in the culture supernatants of TG-induced PEC from LysMCre^+^, MCP-1^flox/flox^ mice activated by LPS for 24 h (Figure 1B). When myeloid cells, especially macrophages, were enriched by incubating PEC in a tissue culture plate at 37°C for 3 h and removing non-adherent cells, almost 100% of the MCP-1 allele was the mutated allele (Figure 1C). These results indicate that the MCP-1 gene was effectively deleted in the myeloid cells of LysMCre^+^, MCP-1^flox/flox^ mice.

### Myeloid-specific MCP-1 deletion did not affect serum MCP-1 concentration in adult mice

**Figure 2:**
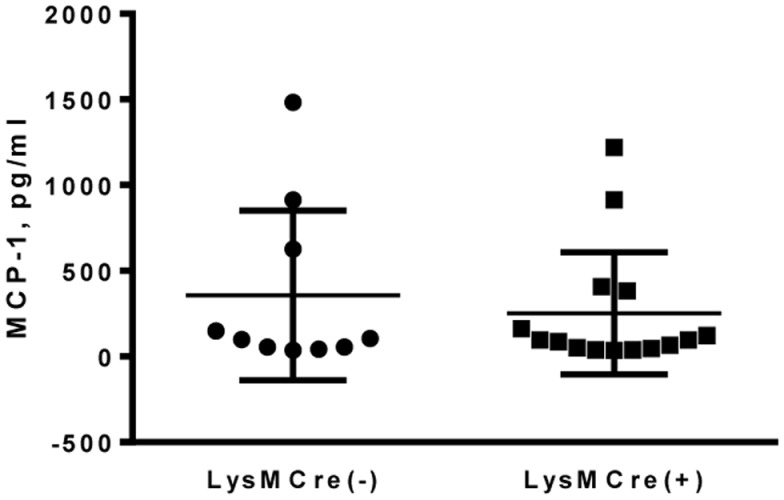
**MCP-1-deficiency in myeloid cells did not affect serum MCP-1 concentrations in normal adult mice**. Serum was collected from 18- to 20-weeks untreated MCP-1^flox/flox^ or LysMCre^+^, MCP-1^flox/flox^ mice and MCP-1 concentration was measured by ELISA.

Previous reports have shown detectable levels of MCP-1 in the sera of healthy human or mice (18, 19). To determine whether MCP-1-deficiency in myeloid cells affects the MCP-1 level in sera, we obtained sera from adult MCP-1^flox/flox^ or LysMCre^+^, MCP-1^flox/flox^ mice and measured MCP-1 concentration. As shown in Figure 2, there was no difference in serum MCP-1 concentration between MCP-1^flox/flox^ and LysMCre^+^, MCP-1^flox/flox^ mice, indicating that myeloid cells do not play a significant role in MCP-1 production under steady state conditions.

### Myeloid-specific MCP-1 deletion had no effect on MCP-1 production or macrophage infiltration in peritonitis

**Figure 3:**
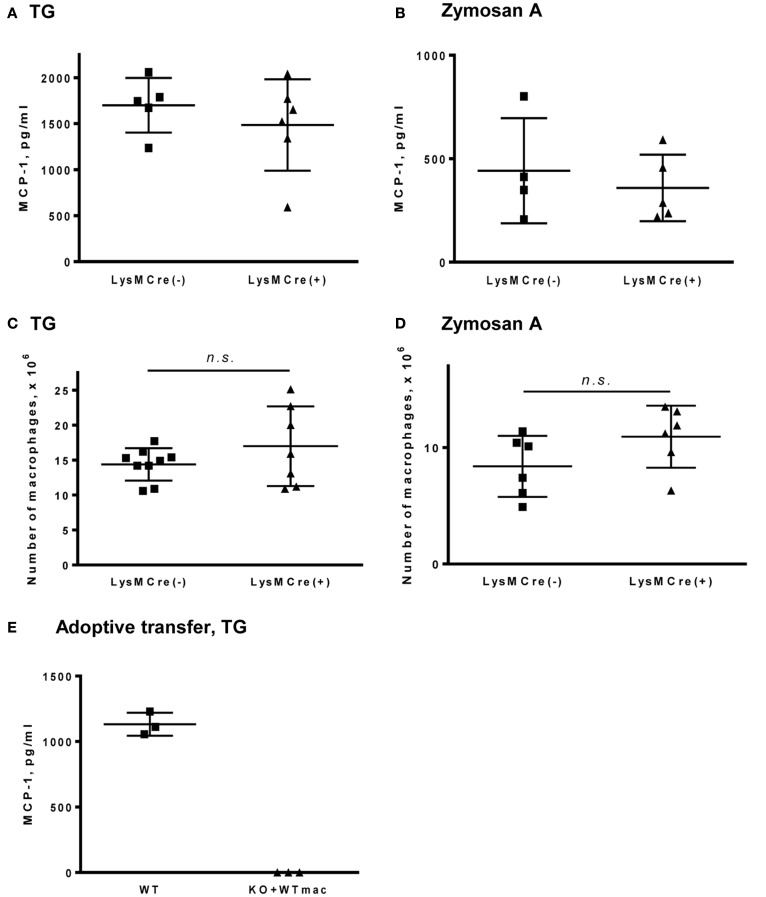
**MCP-1-deficiency in myeloid cells does not affect the concentration of MCP-1 or the accumulation of macrophages in peritoneal fluids in response to TG or zymosan A**. **(A,B)** Peritoneal fluids were collected 4 h after i.p. injection of 1 ml of 3% TG **(A)** or 0.5 ml of 400 μg/ml zymosan A **(B)** and the concentration of MCP-1 in cell-free fluids was measured by ELISA. Data is presented as the mean ± SD obtained from the indicated number of mice. **(C,D)** Mice were i.p. injected with 1 ml of 3% TG **(C)** or 0.5 ml of 400 μg/ml zymosan A **(D)**. Peritoneal cavities were flushed with 5 ml PBS 4 days after injection and the number of macrophages was counted. Data is presented as the mean ± SD. **(E)** Peritoneal resident cells were collected from WT C57BL/6 mice and transferred into the peritoneal cavity of MCP-1 KO mice. WT and MCP-1 KO mice adoptive transferred with WT peritoneal cells were i.p. injected with 1 ml of 3% TG. Peritoneal cavities were flushed with 5 ml PBS 4 h after injection and the concentration of MCP-1 in the peritoneal fluids was measured by ELISA. Data is presented as the mean ± SD obtained from three mice.

To determine whether myeloid cells contribute to MCP-1 production during innate immune responses, we induced peritonitis in MCP-1^flox/flox^ and LysMCre^+^, MCP-1^flox/flox^ mice by i.p injection of TG or zymosan A and examined the concentration of MCP-1 and the number of accumulated macrophages in peritoneal exudates. We previously demonstrated that i.p. injection of TG or zymosan A into normal mice induced the production of MCP-1 which peaked at 4 h and systemic MCP-1 deficiency significantly reduced the accumulation of macrophages into the peritoneal cavity (14). As shown in Figures 3A,B, similar levels of MCP-1 protein were detected in peritoneal exudates of both mouse strains after i.p. injection with either TG or zymosan A. Additionally, there was no difference in the number of macrophages that accumulated in the peritoneal cavity 4 days after induction of peritonitis (Figures 3C,D).

To further evaluate the role of peritoneal resident macrophages in MCP-1 production in TG-induced peritonitis, we adoptively transferred WT resident peritoneal cells into the peritoneal cavities of systemic MCP-1^−/−^ mice, followed by TG-injection. As shown in Figure 3E, there was no detectable level of MCP-1 in the peritoneal exudates of MCP-1^−/−^ mice that received adoptively transferred WT peritoneal resident cells 4 h after TG-injection. These results indicate that myeloid cells are not a major source of MCP-1 during TG- or zymosan A-induced peritonitis.

### Myeloid-specific MCP-1 deletion did not affect MCP-1 production in LPS-induced inflammation in skin air pouch

**Figure 4:**
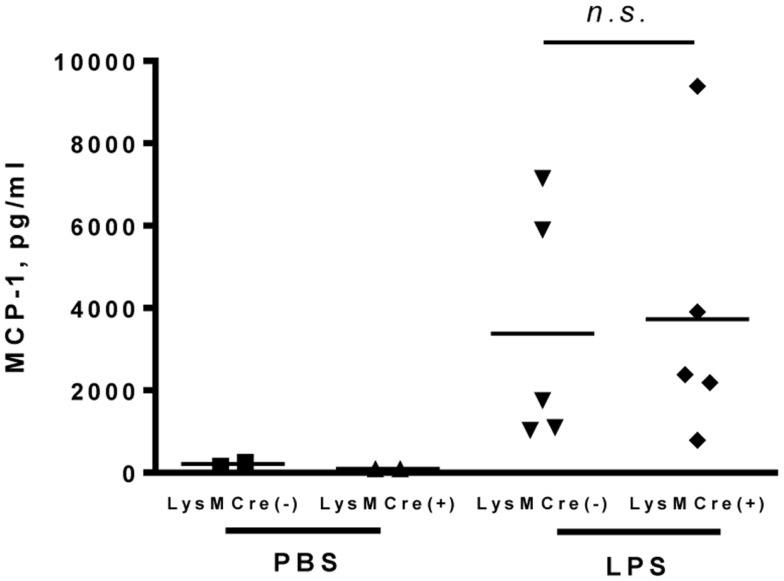
**MCP-1-deficiency in myeloid cells does not alter the production of MCP-1 in response to LPS in air pouches**. Each mouse was given an injection with 1 ml PBS or PBS containing 1 mg LPS into the air pouch. Four hours after injection, air pouches were flushed with 2 ml PBS with heparin. The concentration of MCP-1 in the air pouch fluids was measured by ELISA. Data is presented as the mean ± SD.

It was previously shown that MCP-1 is produced during arthritis in human and in animal models (20, 21). We used an air pouch model as a tool to evaluate the role of myeloid cells in MCP-1 production during arthritis. Since repeated injection of air into subcutaneous connective tissue in the skin results in the formation of a cavity (air pouch) with a lining structure closely resembling synovial tissue, the air pouch model has been used as a convenient model for studying the behavior of synovial lining tissues (22). We injected PBS or LPS (1 mg in 1 ml PBS) into the air pouch and measured MCP-1 concentration in the lavage after 4 h. As shown in Figure 4, only low levels of MCP-1 were detected in the lavage after PBS injection, whereas high levels of MCP-1 were detected in the lavage after LPS injection in both MCP-1^flox/flox^ and LysMCre^+^, MCP-1^flox/flox^ mice with no significant difference between the two strains. These results indicate that, similar to peritonitis, myeloid cells are not a major source of MCP-1 during LPS-induced inflammation in skin air pouch.

### Myeloid-specific MCP-1 deletion did not affect MCP-1 production or macrophage infiltration in LPS-induced lung injury

**Figure 5:**
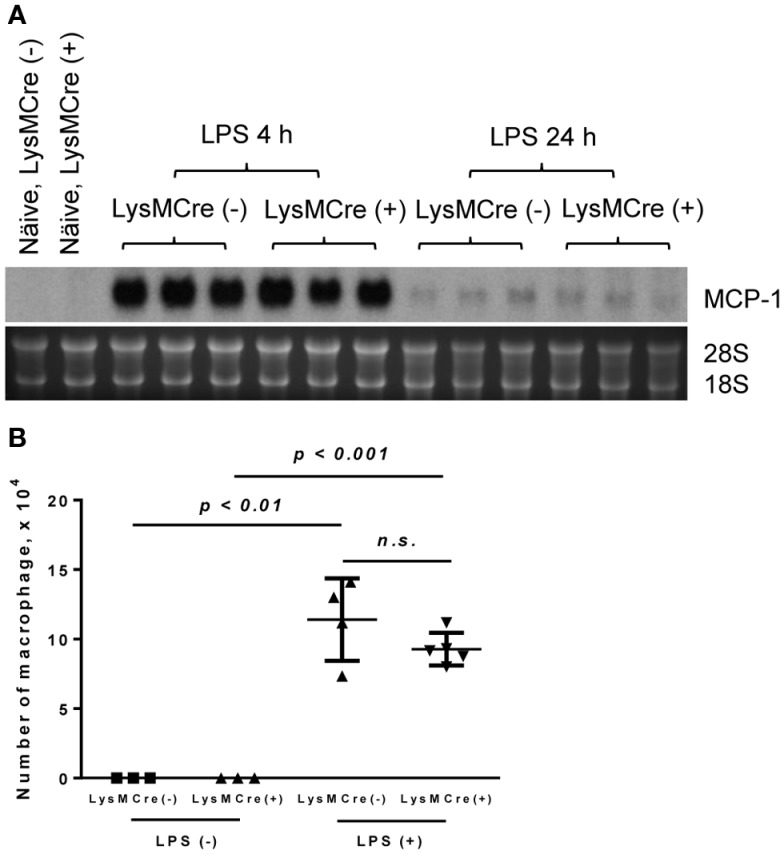
**MCP-1-deficiency in myeloid cells does not alter the production of MCP-1 in LPS-induced lung injury**. Mice were exposed to LPS aerosol (100 or 1000 μg/ml in 10 ml PBS) for 30 min. Four or 24 h after LPS exposure, mice were euthanized by CO_2_ and BALFs were collected. **(A)** The expression of MCP-1 mRNA was examined by Northern blotting. **(B)** The number of macrophages was counted. Data is presented as the mean ± SD.

We next examined LPS-induced lung injury in mice, a model similar to human lung injury that occurs during pneumonia or sepsis. A number of macrophages reside in both alveolar space and lung tissues and these macrophages can be activated in response to LPS to produce MCP-1. We obtained mouse lungs 6 and 24 h after the exposure to LPS and evaluated MCP-1 mRNA expression in whole lung tissue. As shown in Figure 5A, there was no detectable MCP-1 mRNA in normal lung tissue of either MCP-1^flox/flox^ or LysMCre^+^, MCP-1^flox/flox^ mice. After LPS treatment, the expression of MCP-1 mRNA in the lung was readily detectable at 4 h and returned almost to the basal levels by 24 h in both strains. Thus, MCP-1 deletion in myeloid cells did not alter the level of MCP-1 mRNA in LPS-challenged lungs. Accordingly, there was no decrease in the number of macrophages contained in BALFs of LysMCre^+^, MCP-1^flox/flox^ mice 24 h after LPS exposure (Figure 5B).

## Discussion

Macrophages play an important role in the initiation and development of innate immune responses by producing an array of proinflammatory mediators, including cytokines and chemokines (23). Since macrophages produce high levels of MCP-1 in culture *in vitro* and they were often associated with MCP-1 in inflamed tissues, we hypothesized that monocyte/macrophage-specific deletion of the MCP-1 gene might lead to decreased MCP-1 levels at inflammatory sites and subsequent reduction in macrophage accumulation. To test this hypothesis, we generated myeloid cell-specific MCP-1-deficient mice, and evaluated the role of myeloid cells, especially macrophages, in MCP-1 production during innate immune responses. In contrast to our hypothesis, deletion of the MCP-1 gene in myeloid cells had no effects on either MCP-1 production or subsequent macrophage infiltration in three models of innate immune response, indicating that non-myeloid cells are critical in regulating the development of innate immune responses as the primary MCP-1-producing cells.

Sera from healthy human donors contain a wide range of MCP-1 concentrations (18), presumably due to variable degree of stimulation. Sera from normal mice also contain a detectable level of MCP-1 (19). In the present study, myeloid cell-specific MCP-1-deficiency had no effect on the level of serum MCP-1 in naive mice, indicating that MCP-1 is produced by non-myeloid cells and released into serum. We previously examined the cellular sources of MCP-1 in human atherosclerotic lesions by immunohistochemistry. Although foam cells infiltrating the aorta were strongly positive for MCP-1, ECs were also positive. Furthermore, aortic ECs from an individual with no apparent atherosclerosis were positive for MCP-1 (24). Thus, non-myeloid cells, likely ECs, appear to be the source of MCP-1 found in sera of human and mice.

To determine the cellular source of MCP-1 in innate immune responses, we utilized two mouse peritonitis models, induced by TG or zymosan A. The MCP-1 concentration in the exudates of myeloid cell-specific MCP-1-deficient mice was not decreased in either peritonitis model, indicating that non-myeloid cells are the primary source of MCP-1. The role of peritoneal resident macrophages and mast cells in chemokine production in acute inflammation was previously investigated in mouse peritonitis models (25). Depletion of either macrophages or mast cells had no effect on the production of MCP-1 or the neutrophil attracting chemokine KC after TG-injection into WT mice. Removal of either macrophages or mast cells resulted in attenuation of neutrophil infiltration into the peritoneal cavity of WT mice without affecting the levels of MCP-1 and KC in response to intraperitoneal administration of LPS; however, depletion of resident mast cells inhibited neutrophil accumulation as well as MCP-1 and KC production in response to zymosan, suggesting that mast cells may be the primary source of MCP-1 in peritonitis caused by certain stimuli, such as zymosan. Mesothelial cells form a monolayer that lines the pleural, peritoneal, and pericardial cavities as well as internal organs (26), and produce chemokines, including KC and MCP-1, in response to ligands of Nod1 or TLRs (27). Therefore, mesothelial cells may be a potential cellular source of MCP-1 in our study. These findings support our conclusion that non-myeloid cells in the peritoneal cavity, but not resident peritoneal macrophages, are the major source of MCP-1 in innate immune responses.

In addition to the peritonitis models, we used a LPS-induced lung injury model to identify the source of MCP-1 in innate immune responses. A previous study indicated that MCP-1 produced by alveolar macrophages mediated systemic inflammation caused by acute alveolar hypoxia, using rats in which alveolar macrophages were depleted by airway instillation of clodronate-containing liposomes (28). In our study using genetically engineered mice, we demonstrate that non-myeloid cells were the primary source of MCP-1 in LPS-induced lung injury. Additionally, intraperitoneal injection of LPS in both MCP-1^flox/flox^ and LysMCre^+^, MCP-1^flox/flox^ mice induced high levels of MCP-1 mRNA in the lung of both strains (data not shown). Thus, non-myeloid cells in the lung, such as bronchoalveolar cells which have the capacity to express MCP-1 (29), likely are the cellular source of MCP-1 to regulate the accumulation of macrophages in this model.

Myeloid cells, such as macrophages, have been thought to be the major contributor to the development of innate immune responses by releasing a variety of proinflammatory mediators, including chemokines. However, our study has clearly demonstrated that non-myeloid cells play a previously unappreciated role in the development of innate immune response by acting as the major producer of MCP-1. While we did not observe an effect of myeloid-specific MCP-1 deletion in these models, there is a possibility that effects may be observed in other models of disease. This may be especially true in chronic models of inflammation, in cases of monocyte-macrophage infection with intracellular pathogens, such as *L. monocytogenes* or in tumor-associated macrophages in cancer. Therefore, our MCP-1^flox/flox^ mice will be a great tool to investigate the relative contribution of different cell types to the development of immune responses and also cancer in which MCP-1 contribute to its progression.

## Author Contributions

Teizo Yoshimura performed the majority of experiments, and wrote the paper; Carole Galligan, Munehisa Takahashi, and Lino Tessarollo contributed to the generation of MCP-1 floxed mice; Keqiang Chen helped in experimental design; Mingyong Liu performed ELISA; Ji Ming Wang helped in experimental design and reviewed the paper.

## Conflict of Interest Statement

The authors declare that the research was conducted in the absence of any commercial or financial relationships that could be construed as a potential conflict of interest.
